# PGRMC1 Promotes the Development of Cervical Intraepithelial Neoplasia in HPV-Positive Patients

**DOI:** 10.3390/biomedicines13102454

**Published:** 2025-10-09

**Authors:** Wen Lai, Shuyu Liu, Tianming Wang, Min Gong, Qiaoling Liu, Ling Ling, Jianquan Chen

**Affiliations:** 1 Department of Obstetrics and Gynecology, The Affiliated Jiangning Hospital with Nanjing Medical University, Nanjing 211199, China; 2 The First Clinical Medical College, Nanjing Medical University, Nanjing 211166, China; 3 Central Laboratory, Translational Medicine Research Center, The Affiliated Jiangning Hospital with Nanjing Medical University, Nanjing 211199, China

**Keywords:** PGRMC1, cervical cancer, VIM, cervical intraepithelial neoplasia

## Abstract

**Background/Objectives**: Persistent human papillomavirus (HPV) infection is the leading cause of cervical intraepithelial neoplasia (CIN), a known precursor to cervical squamous carcinoma. While progesterone receptor membrane component 1 (PGRMC1) has been implicated in various cancers, its specific role in cervical carcinogenesis has remained uncertain. This study aimed to elucidate the function of PGRMC1 in the progression of CIN. **Methods**: Bioinformatics techniques were employed to assess the expression levels of PGRMC1 in cervical cancer tissues and to investigate its correlation with patient prognosis. To explore the functional role of PGRMC1, we manipulated its expression in the cervical cancer cell line HeLa using siRNA. Subsequently, we evaluated cell migration via the scratch assay, and invasion through the Transwell assay. We employed mass spectrometry to identify proteins interacting with PGRMC1 and confirmed these interactions using co-immunoprecipitation (co-IP). Further co-IP experiments were conducted to pinpoint the specific binding sites of these protein interactions, and immunofluorescence staining was utilized to observe the spatial distribution of interacting proteins within the cells. The phosphorylation status of VIM was further confirmed by WB. At the clinical level, we collected cervical biopsy specimens from HPV-positive patients and verified the expression patterns of PGRMC1 and VIM using immunohistochemical staining in cervical squamous cell carcinoma (CSCC) tissues. **Results**: We discovered a correlation between progressively increasing PGRMC1 expression and the severity of CIN as well as a poor prognosis. Knockdown of PGRMC1 resulted in the inhibition of migration and invasion capabilities in cervical cancer cells. Furthermore, PGRMC1 was found to physically interact and colocalize with Vimentin (VIM). Notably, PGRMC1 knockdown specifically increased phosphorylation at the Ser-39 residue of VIM. **Conclusions**: Our findings suggest that PGRMC1 facilitates CIN progression by binding to VIM and suppressing Ser-39 phosphorylation, thereby promoting the migration and invasion of cervical carcinoma cells. This study enhances our understanding of PGRMC1’s role in CIN progression and lays an experimental foundation for targeted therapeutic approaches to cervical squamous carcinoma.

## 1. Introduction

Cervical cancer ranks as the fourth most prevalent malignant tumor among women globally, posing a significant public health challenge that affects women’s well-being [1]. Data from 2020 reveal approximately 604,127 new cases of cervical cancer and 341,831 related deaths, underscoring the disease’s substantial burden [2]. Histologically, cervical cancer is classified primarily according to pathomorphologic characteristics and can be categorized into subtypes such as squamous cell carcinoma, adenocarcinoma, and adenosquamous carcinoma. Cervical squamous cell carcinoma (CSCC) constitutes roughly 70% to 80% of all cervical cancer cases, making it the most prevalent pathological type [3]. Persistent infection with high-risk human papillomavirus (HPV) is identified as the primary cause of CSCC. Such persistent HPV infection can lead to cervical squamous intraepithelial lesions, which are further classified into low-grade squamous intraepithelial lesions (LSIL) and high-grade squamous intraepithelial lesions (HSIL) based on the depth and extent of the lesions within the cervical tissue [4]. Epidemiological studies indicate that approximately 60% of LSIL cases resolve spontaneously. However, some cases progress to invasive carcinoma due to persistent HPV infection, advancing to HSIL [5]. HSIL, a precancerous lesion of cervical cancer, is often associated with persistent infection by high-risk HPV types and may eventually develop into squamous cell carcinoma of the cervix if not promptly addressed. Currently, the treatment of early-stage cervical cancer relies on surgery, but some patients remain at risk of recurrence. Therefore, gaining a deeper understanding of the mechanisms underlying cervical cancer (CC) development and identifying key therapeutic targets are crucial.

Progesterone receptor membrane component 1 (PGRMC1) is a key member of the Membrane-Associated Progesterone Receptor (MAPR) family, encoded by the PGRMC1 gene located on the X chromosome. This protein features a cytochrome b5-like heme/sterol binding domain and plays a pivotal role in numerous cellular and tissue functions, including cytochrome P450 metabolism, heme homeostasis, carcinogenesis, drug resistance, regulation of female reproduction, and neural function regulation [6]. As a type of membrane receptor, PGRMC1 swiftly responds to progesterone signaling and modulates intracellular signaling processes, notably through the activation of the Ras signaling pathway, among other mechanisms [7]. PGRMC1 has demonstrated a pro-carcinogenic effect in lung, colon, and thyroid cancers, where it can promote tumorigenesis and progression. Notably, in the plasma of lung cancer patients, PGRMC1 expression is significantly elevated, making it a promising new therapeutic target for lung cancer [8]. In breast cancer, PGRMC1 is markedly overexpressed and has been identified as a novel biomarker for characterizing estrogen receptor status and tissue hypoxia [9]. Additionally, in ovarian cancer, stable transfection with PGRMC1-specific siRNA significantly inhibits the growth of transplanted tumors, highlighting its potential as a therapeutic target [10]. However, the role of PGRMC1 in the development and progression of cervical cancer, especially cervical intraepithelial neoplasia (CIN), has not been fully investigated.

In this study, we intended to collect clinical tissue samples and utilize a cervical cancer cell line (HeLa) in vivo and in vitro to study the expression of PGRMC1 in cervical squamous intraepithelial lesion and its possible mechanism. We found that PGRMC1 binds to and regulates VIM phosphorylation to promote cervical squamous intraepithelial lesion progression. Our study expands the understanding of PGRMC1’s role in cervical carcinogenesis and provides an experimental basis for targeted therapy of cervical cancer.

## 2. Materials and Methods

### 2.1. Clinical Tissue Samples

The clinical specimens used in this study were obtained from the remaining portion of HPV-positive patients’ cervix biopsies sent for pathology at Jiangning Hospital in Nanjing, China, and were pathologically graded according to the colposcopic Reid scoring criteria [11]. Finally, they were divided into four groups, including the Cervicitis group, the LSIL group, the HSIL group, and the CSCC group. All clinical samples were obtained from patients who underwent colposcopy at the Obstetrics and Gynecology outpatient clinic of Nanjing Jiangning Hospital from September 2022 to December 2024, and the specimens obtained were the remaining portions of the subjects’ pathology slides. All subjects signed an informed consent form, and the project was examined and approved by the Ethics Committee of Nanjing Jiangning Hospital (Ethics Approval Number: No. 2021-03-020-K01). Patient characteristics was described in Table 1.

### 2.2. Cell Culture, Passaging, and Grouping

HeLa cells and SiHa cells were purchased from HyCyte (#TCH-C326, Suzhou, China) and were cultured in DMEM (Biochannel, Nanjing, China) supplemented with 10% fetal bovine serum (FBS, Biochannel, China) in a humidified atmosphere containing 5% CO_2_ at 37 °C. In the experiment, PGRMC1 siRNA or control siRNA were constructed by tsingke (Beijing, China) and then they were transfected into HeLa cells using Lipomaster 3000 Transfection Reagent (# TL301-01, Vazyme, Nanjing, China). The siRNA sequences were listed in Table 2.

### 2.3. Antibody Preparation

Primary antibodies against PGRMC1 (# A5619), VIM (# A19607), ACTB (# AC0260), MMP2 (# A19080), HA-Tag (# AE124), DDDDK-Tag (# AE121), VIM-S39 (# AP0806), VIM-S56 (# AP0804), VIM-S83 (# AP0799) as well as secondary antibody goat anti-rabbit IgG (# AS014) were from Abclonal (Wuhan, China). Primary antibodies against MMP9 (# HA722106) was purchased from HUABIO (Hangzhou, China).

### 2.4. Bioinformatics Analysis

Prognostic survival data were obtained from the Kaplan–Meier Plotter database (www.kmplot.com). Based on the mean expression value of PGRMC1 in the cervical cancer tissue microarray, the samples were divided into two groups (high expression group and low expression group). The overall survival (OS), distant metastasis free survival (DMFS), post progression survival (PPS), and recurrence free survival (RFS) of patients with cervical cancer were analyzed to study their correlation with the expression of PGRMC1. Data on PGRMC1 expression and the correlation of various clinicopathological parameters of PGRMC1 with cervical cancer were obtained from the UALCAN database (www.ualcan.path.uab.edu). Mining of genes co-expressed with PGRMC1 in cervical cancer was performed using data from the LinkedOmics database, followed by enrichment analysis using the Micro Bioinformatics Platform (www.bioinformatics.com.cn). We defined *p* < 0.05 and FDR < 0.25 as a significantly enriched gene set, and obtained 24,776 genes co-expressed with PGRMC1 in cervical cancer. The data were presented in the form of heat maps, showing the top 50 genes with positive or negative correlations.

### 2.5. Total RNA Isolation and Real-Time Fluorescence Quantitative PCR Analysis

Total RNA was extracted by TRIzol (# 15596018, Invitrogen, Carlsbad, CA, USA) and was then reverse transcribed into cDNA using HiScript II One Step RT-PCR Kit (# P611-01, Vazyme, Nanjing, China). The cDNA was obtained and subjected to qPCR experiments with ChamQ SYBR qPCR Master Mix (# Q341-02, Vazyme, China). The primers were listed in Table 3.

### 2.6. Western Blot Analysis (WB)

HeLa cells were lysed using RIPA buffer, supplemented with a protease inhibitor, to prepare protein samples. Subsequently, these protein samples were separated using a precast gel (Model # M00669, GenScript, Nanjing, China) and then transferred onto PVDF membranes. The membranes were blocked and incubated overnight at 4 °C with the primary antibody, diluted at a ratio of 1:1000. The following day, the membrane was rinsed three times with Western washing solution (Product # P0023C6, Beyotime, Shanghai, China). Next, the secondary antibody (diluted at 1:5000) was added and incubated on a shaker at room temperature for 1 h. The membranes were then treated with ECL luminescent solution (Product # P0018M, Beyotime, Shanghai, China), and the bands were visualized using a chemiluminescent imaging system.

### 2.7. Immunofluorescence Staining

The cells were grown on coverslips in a 12-well plate with a cell coverage rate of about 50%. After treatment, the cells were immobilized with 4% paraformaldehyde for 10 min, permeabilized with 0.2% TritonX-100 at room temperature for 10 min, and followed by a block with 5% BSA for 30 min. Then the 12-well plate was added the primary antibody (used at 1:100 dilution) for incubation in a wet dark box at room temperature. After incubation, primary antibody was removed. The cells were washed with PBS twice and incubated with secondary antibody for 1 h. After incubation, secondary antibody was removed. The cells were washed with PBS twice and incubated with DAPI (# D3571, Invitrogen, USA) for nuclear staining. Images were captured by confocal microscopy and visualized with ZEN software 2.3 version.

### 2.8. Cell Scratching Experiment

HeLa cells or SiHa cells (used at a density of 6 × 10^4^ cells per well) were grown in 6-well culture plate, of which 5 parallel horizontal lines were drawn on the back. According to the pre-marked line, uniform scratches were made vertically against the plate cover of the wells. The marking line on the bottom of the plate was erased, and the scratched area was photographed under an inverted microscope to ensure that the scratches were centered, the edges were perpendicular and the background conditions were consistent in all wells, and the samples were taken and photographed at the time points of 0 h (immediately after the scratches), 6 h, 12 h, and 24 h, respectively. Mark the scratch edges using image J software 1.53t version, and then calculate the area between the two cell edges at 0 h and 24 h. Convert it into the gray values and calculate the migration rates of cells in different groups.

### 2.9. Transwell Cell Invasion Assay

HeLa cells were grown in a 6-well culture plate at a concentration of 2 × 10^5^ cells per well. The next day, the cells were transfected with PGRMC1 siRNA or negative siRNA for 24 h, and then the cells were digested and resuspended. For invasion experiments, matrigel matrix gel (Corning, NY, USA) was diluted with ice-cold serum-free medium, and was uniformly encapsulated in the basement membrane of the upper chamber of the Transwell (Corning, NY, USA). The cell suspension was added into the upper chamber of the pre-coated matrigel chambers, and the complete medium was added to the lower chamber. For migration assays, cell suspensions were added to Transwell chambers without matrigel matrix gel. The chambers were taken out at indicated time, washed and fixed in methanol for 20 min. After a crystal violet staining, the upper layer cells were wiped off and the cell number was counted under the microscope. The invasive ability is reflected by comparing the number of cells passing through the chambers in different groups.

### 2.10. Coimmunoprecipitation (Co-IP)

HEK293T cells were co-transfected with FLAG-tagged *PGRMC1* plasmid and HA-tagged VIM or truncated proteins plasmid for 48 h. Then the samples were washed three times with ice-cold PBS and lysed with IP buffer (RIPA Buffer with protease inhibitor and phosphatase inhibitor) in an ice bath for 30 min. The lysate was centrifuged at 12,000× *g* for 10 min at 4 °C, and the supernatant was collected and incubated overnight with Anti-DYKDDDDK (Flag) Affinity Gel (# HAK21024, HUABIO, Hangzhou, China) at 4 °C. The gel was washed more than three times, added 5 × SDS-PAGE Loading Buffer and boiled at 100 °C for 10 min. The samples were then assayed by WB validation.

### 2.11. Statistical Analysis

All data are presented as the mean ± standard deviation (SD). Student’s *t*-test was employed for comparisons between two independent sample groups. For single-factor comparisons among multiple groups, a one-way analysis of variance (ANOVA) was utilized, while a two-way ANOVA was used for two-factor comparisons among multiple groups. A *p*-value of less than 0.05 was considered statistically significant.

## 3. Results

### 3.1. PGRMC1 Expression in the Process of CIN-CSCC Transition

**Figure 1:**
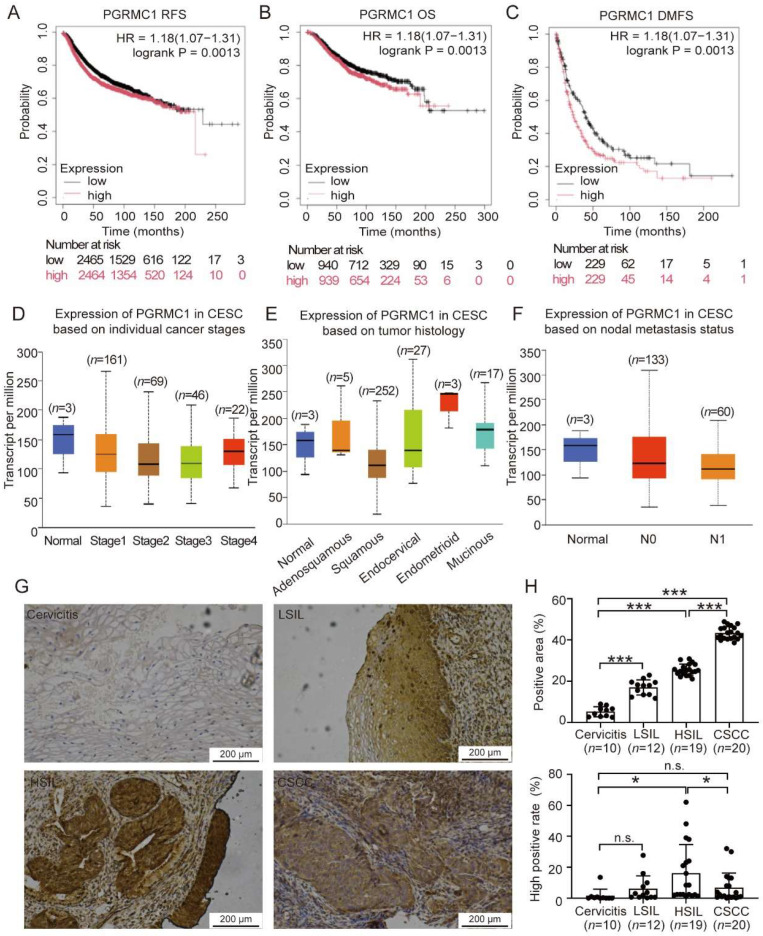
Expression of PGRMC1 in cervical tissues and its verification in tissues (**A**) recurrence free survival, RFS; (**B**) overall survival, OS (**C**) distant metastasis-free survival, DMFS (**D**) the RNA expression levels of PGRMC1 based on cancer stage (**E**) the RNA expression levels of PGRMC1 based on tumor subtype (**F**) the RNA expression levels of PGRMC1 based on lymphatic metastasis (**G**) immunohistochemical staining of PGRMC1 in cervical tissues (n = 5). (**H**) The positive area and high positive rate of PGRMC1 expression in cervical tissues related to G. * = *p* < 0.05, *** = *p* < 0.001, n.s.= not significant. Data are mean ± SD.

First, we evaluated the prognostic survival of cervical cancer patients using the Kaplan–Meier Plotter, based on data from the TCGA database. Patients were categorized into two groups according to the mean expression level of PGRMC1 in cervical cancer tissue microarrays: a high-expression group (expression above the mean) and a low-expression group (expression below the mean). Kaplan–Meier survival analysis revealed that patients with high PGRMC1 expression exhibited significantly lower recurrence-free survival (RFS) (Figure 1A) and overall survival (OS) (Figure 1B) compared to those with low PGRMC1 expression. Additionally, distant metastasis-free survival (DMFS) (Figure 1C) was markedly reduced in the high-expression group, with all differences reaching statistical significance. These findings suggest that elevated PGRMC1 expression serves as a poor prognostic indicator in cervical cancer patients. Next, we utilized the UALCAN database to investigate PGRMC1 expression and its association with various clinicopathological parameters, including gender, ethnicity, age, perimenopausal status, and tissue type. Our analysis revealed that PGRMC1 expression was generally lower in cervical cancer tissues compared to normal and cervical tissues. Furthermore, PGRMC1 expression was significantly correlated with cancer stage, tumor tissue subtype, and lymphatic metastasis status (Figure 1D–F). However, we noted a limitation in the TCGA database: the sample size for normal cervical tissue was extremely small, comprising only three specimens. Obtaining normal cervical tissue and extracting RNA from it is challenging. Moreover, the presence of non-cervical epithelial tissue, such as glandular tissue, in the samples may interfere with the accurate detection of target gene expression. Given that immunohistochemical (IHC) staining can spatially distinguish target gene expression, we proceeded to perform IHC staining to validate PGRMC1 expression in cervical tissues.

To further validate the expression of PGRMC1 in cervical cancer, we gathered specimens from the residual portions of cervical biopsies obtained from HPV-positive patients. These biopsies were sent for pathological examination to Jiangning Hospital in Nanjing, China, between September 2022 and December 2024. The clinical tissue samples were categorized into four groups based on the degree of cervical histopathology: Cervicitis, LSIL, HSIL, and CSCC. Random selections from each of these four groups were subjected to immunohistochemistry. Images were captured using microscopy, and the positive rate was determined with the IHC profiler plugin in ImageJ. Both the positive area and positive rate were utilized to assess the protein expression of PGRMC1 in the tissues of Cervicitis, LSIL, HSIL, and CSCC. The results indicated a gradual intensification of PGRMC1 staining from the Cervicitis group to the LSIL group, then to the HSIL group, with the CSCC group also displaying strong staining (Figure 1G). To further verify the expression characteristics of PGRMC1 in cervical lesion tissues, we quantified the positive area and high positive rate of the IHC images using ImageJ software, 1.53t version. The findings revealed a progressive increase in the positive area of PGRMC1 staining from the Cervicitis group to the CSCC group, suggesting that PGRMC1-positive cells gradually dominated the cervical tissue during the transition from CIN to CSCC. Furthermore, compared to the Cervicitis group, the high positive rate of PGRMC1 protein in HSIL tissues was significantly elevated. However, the high positivity rate in CSCC tissues was significantly lower than that in the HSIL group, with no significant difference observed between the Cervicitis group and CSCC group (Figure 1H). These findings indicate that PGRMC1 expression increases during CIN development.

Taken together, the above experimental results indicated that during the process of cervical precancerous lesions, the expression level of PGRMC1 gradually increases and it is an adverse prognostic factor for cervical cancer.

### 3.2. Silencing PGRMC1 Significantly Reduces the Migration and Invasion of CSCC Cells

**Figure 2:**
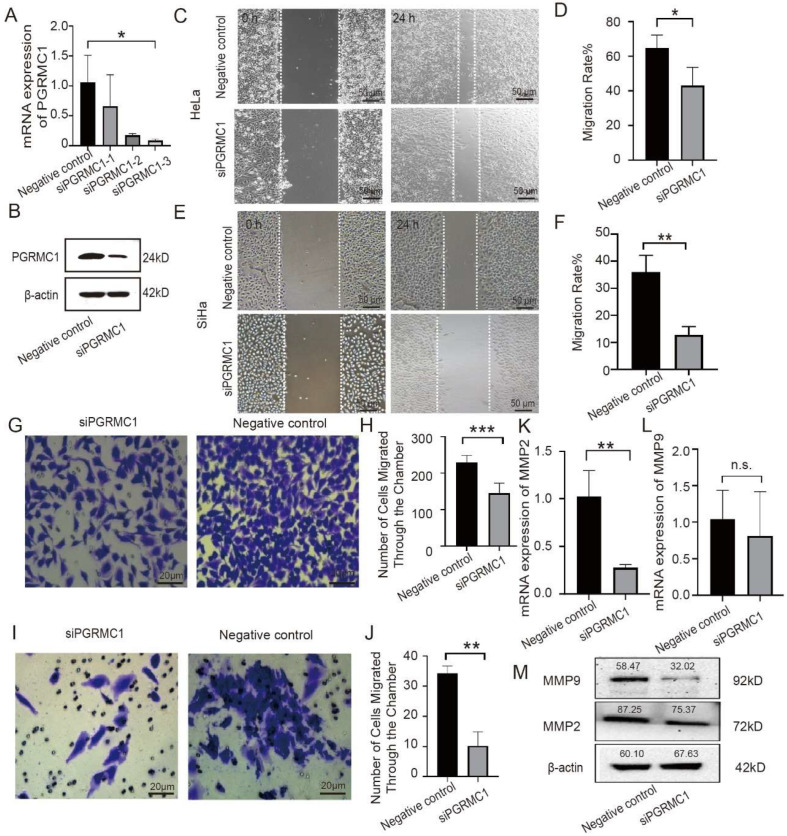
Effects of PGRMC1 expression on the migration and invasion capabilities of CSCC cell lines. (**A**) qPCR was used to analyze mRNA expression of PGRMC1 in HeLa cells transfected with PGRMC1 siRNA or negative control siRNA; (**B**) Western blot was used to analyze protein levels of PGRMC1 in HeLa cells transfected with PGRMC1 siRNA or negative control siRNA, the data are normalized to the β-actin control; (**C**,**D**) scratch test of HeLa cells transfected with PGRMC1 siRNA or negative control siRNA; (**E**,**F**) scratch test of SiHa cells transfected with PGRMC1 siRNA or negative control siRNA; (**G**,**H**) Transwell test of HeLa cells transfected with PGRMC1 siRNA or negative control siRNA to show the ability of cell migration; (**I**,**J**) Transwell test of HeLa cells transfected with PGRMC1 siRNA or negative control siRNA to show the ability of cell invasion; (**K**) qPCR was used to analyze mRNA expression of MMP2; (**L**) qPCR was used to analyze mRNA expression of MMP9; (**M**) Western blot was used to analyze protein levels of MMP2, MMP9 and β-actin in HeLa cells transfected with PGRMC1 siRNA or negative control siRNA, the data are normalized to the β-actin control. All data are from three independent experiments. * = *p* < 0.05, ** = *p* < 0.01, *** = *p* < 0.001, n.s. = not significant. Data are mean ± SD.

To study the effect of PGRMC1 on cervical cancer, we transfected HeLa cells with siRNA to knock down PGRMC1 so as to verify its effect on the biological function. We first transfected positive control siRNA (GAPDH) into the cells to evaluate the transfection efficiency. We diluted the siRNA premix with Opti-MEM medium and mixed it with Lipo3000 reagent. After transfection for 6 h, transfection reagent was removed, fresh complete medium was added, and the HeLa cells were continued to culture for 48 h. We thus confirmed that the degree of transcriptional level inhibition of GAPDH was greater than 70% by qPCR, indicating that this method has a relatively good knockdown efficiency. Using the same method, we transfected PGRMC1-specific siRNA into HeLa cells. The qPCR results showed that: Although all three siRNA reduced the expression of PGRMC1, siPGRMC1-3 had the strongest effect (the degree of transcriptional level inhibition of PGRMC1 was greater than 80%). Eventually, we chose siPGRMC1-3 for the subsequent experiments (Figure 2A). Subsequently, we utilized Western blot assay to determine the protein expression of the two groups of cells, and the results showed that the protein expression of siPGRMC1 group was significantly lower than that of the control group (Figure 2B).

After that, we verified the effect of siPGRMC1 on cell migration using cell scratch assay and Transwell cell migration assay. The results of the scratch experiment showed that, at the same time, the migration distance of siPGRMC1 group was shorter than that of the control group (Figure 2C), and the migration rate of the siPGRMC1 group was significantly lower than the control group (Figure 2D). A similar trend was also found in SiHa cells (Figure 2E), the migration rate of the siPGRMC1 group was significantly lower than the control group (Figure 2F). In addition, the results of Transwell cell migration experiment showed that fewer cells passed through the chambers in the siPGRMC1 group than in the control group (Figure 2G), and the control group’s number of migrating cells was significantly higher than the siPGRMC1 group (Figure 2H). The above results suggested that interfering with PGRMC1 could inhibit the migration ability of HeLa cells.

Next, a second Transwell cell invasion assay was conducted to investigate the impact of PGRMC1 on the invasive capacity of cervical cancer epithelial cells. Specifically, HeLa cells were transfected with either PGRMC1 siRNA or a control siRNA. After 24 h, the number of cells that traversed the chambers was quantified using statistical methods. The experimental results revealed that the siPGRMC1 group exhibited a significantly lower number of cells crossing the chambers compared to the control group (Figure 2I). Furthermore, a detailed count of migrated cells confirmed that the siPGRMC1 group had a markedly reduced number of invasive cells relative to the control group (Figure 2J). These findings collectively suggest that interfering with PGRMC1 expression suppresses the invasive ability of HeLa cells.

We then investigated the impact of PGRMC1 on the expression of genes associated with cell migration, specifically MMP2 and MMP9. To achieve this, we transfected HeLa cells with siPGRMC1, thereby establishing an siPGRMC1 group alongside a control group. The qPCR analysis revealed that the mRNA expression level of MMP2 was significantly reduced in the siPGRMC1 group compared to the control group (Figure 2K). Although the mRNA expression level of MMP9 was also lower in the siPGRMC1 group, the difference was not statistically significant (Figure 2L). Further, Western blot analysis demonstrated that the protein expression levels of both MMP2 and MMP9 were lower in the siPGRMC1 group than in the control group (Figure 2M). Collectively, these findings suggest that suppressing PGRMC1 expression influences the migratory capacity of cervical cancer cells.

Based on the above experimental results, we draw the conclusion that inhibiting the expression of PGRMC1 can suppress the expression of proteins related to the migration and invasion of cervical cancer cells, thereby inhibiting the migration and invasion ability of the cells.

### 3.3. PGRMC1 Binds to VIM

**Figure 3:**
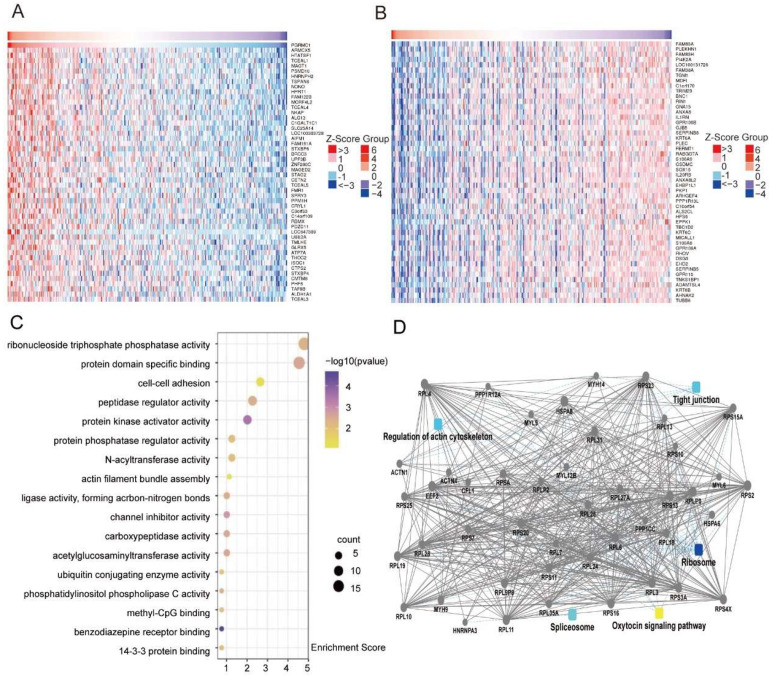
Screening of co-expressed genes and interacting proteins of PGRMC1. (**A**) co-expressed genes of PGRMC1 in cervical cancer were analyzed using the LinkedOmics database. Genes with *p* < 0.05 and false discovery rate (FDR) < 0.25 were defined as significantly enriched, yielding 24,776 co-expressed genes. The heatmap displays the top 50 positively correlated genes. (**B**) The heatmap displays the top 50 negatively correlated genes. (**C**) Bubble plot of GO and KEGG enrichment analyses for PGRMC1 co-expressed genes. (**D**) Protein–protein interaction network of PGRMC1, with five significantly associated pathways highlighted.

The positive rate of PGRMC1 protein expression in cervical tissues of different pathologic types was analyzed by using LinkedOmics database to search for the co-expressed genes of PGRMC1 in cervical cancer. There were 24,776 genes were found to be co-expressed in cervical cancer, and the top 50 genes that were positively or negatively correlated were plotted in the heatmap (Figure 3A,B). GO and KEGG enrichment analyses of the co-expressed genes showed that they were mainly enriched in the processes of cell adhesion and actin fiber bundle assembly, suggesting that PGRMC1 may be involved in the regulation of cell transduction pathway in cervical cancer (Figure 3C).

**Figure 4:**
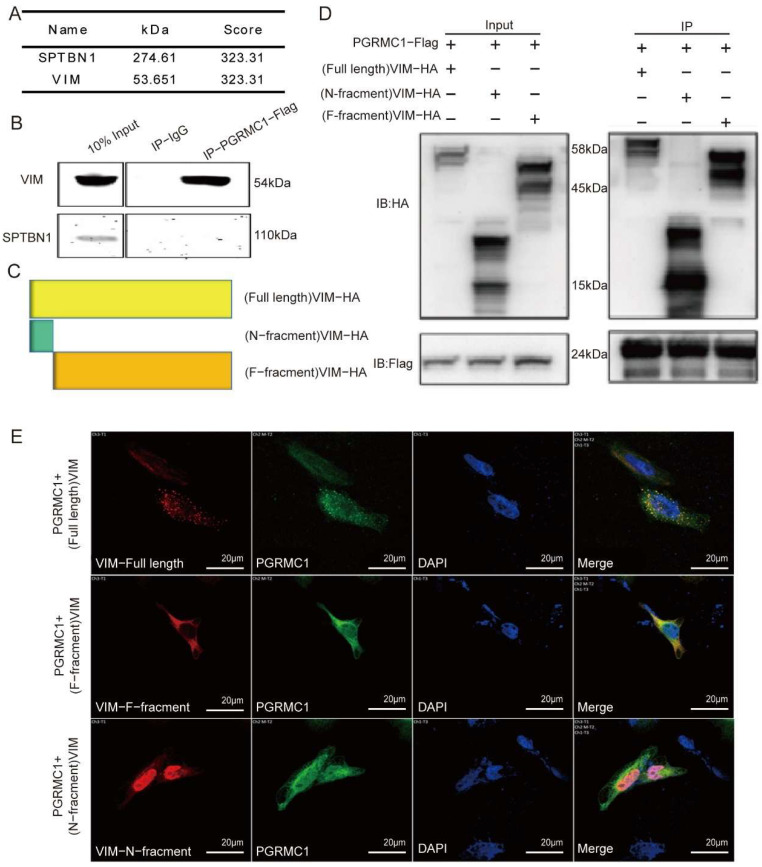
Validation of PGRMC1 binding to VIM. (**A**) protein identification table related to PGRMC1 interactome using mass spectrometry (**B**) Co-IP assays were used to verify the interaction of PGRMC1 with SPTBN1 and VIM. HEK293T cells were transfected with FLAG-PGRMC1 plasmid and were IP with anti-FLAG antibody or IgG control; (**C**) map of full length of VIM and the truncated (F and N) fractions; (**D**) Co-IP assays were used to verify the interaction of PGRMC1 with VIM and the truncated (F and N) fractions. HEK293T cells were co-transfected with FLAG-PGRMC1 plasmid and full-length VIM plasmid or the truncated fraction plasmids; (**E**) Immunofluorescence staining of PGRMC1(green), VIM and the truncated (F and N) fractions (red) to show colocalization in HeLa cells.

Given that PGRMC1 can influence the function of cervical cancer cells at the cellular level, we conducted a mass spectrometry analysis to screen a total of 246 proteins associated with PGRMC1. Subsequently, we constructed a protein interaction network through bioinformatic analysis (Figure 3D), which revealed that five pathways were closely linked to PGRMC1: tight junctions, ribosomes, the cathepsin signaling pathway, splicing bodies, and the cytoskeleton. After evaluating and identifying the associated proteins, we selected two candidate proteins—SPTBN1 and Vimentin (VIM)—for protein interaction validation (Figure 4A). To achieve this, 293T cells were transfected with a FLAG-labeled PGRMC1 plasmid for 48 h. We then verified the interactions between PGRMC1 and the candidate proteins using immunoprecipitation (Co-IP) experiments. The Co-IP results demonstrated that PGRMC1 bound to VIM; however, no SPTBN1 band was detected in the Co-IP samples transfected with the PGRMC1 plasmid (Figure 4B).

To further explore the domain where VIM bind with PGRMC1, 2 more plasmids were constructed: (N-fragment) VIM-HA and (F-fragment) VIM-HA (Figure 4C). Then PGRMC1 was co-transfect with the following three VIM plasmids: full length VIM, N-fragment, F-fragment. And the corresponding bands were detected after immunoblotting with the corresponding label antibodies; immunoprecipitation experiments were carried out based on this. The results showed that: after IP with Flag and immunoblotting with HA antibody, a clear band appeared in group 1 (lane 1), and after comparison, the target band was around 58 kD, which was basically in line with the size of the protein of VIM (54 kD); a clear band appeared in group 2 (lane 2), and after comparison, the target band was around 15 kD, which was in line with the size of the protein of N-fragment (15 kD); a clear band appeared in group 3 (lane 3), and after comparison, the target band was around 45 kD; the target band was around 45 kD; the target band was around 45 kD. In group 3 (3 lanes), a clear band appeared, and after comparison, the target band was around 45 kD, which was consistent with the protein size of F-fragment (45 kD); after immunoblotting with Flag antibody, a clear band appeared in all 3 groups, and after lateral comparison of pre-stained Marker, the size of the band was around 24 kD, which was consistent with the size of Flag (Figure 4D). Therefore, the results indicated that in 293 cells, both VIM-HA protein and its N-fragment and F-fragment interacted with PGRMC1-FlAG protein.

In order to investigate the Spatial distribution of PGRMC1 and VIM, we co-transfected PGRMC1 plasmid with VIM plasmid or its truncated plasmid, and then performed immunofluorescence staining and photographed with Laser copolymer microscope. The results showed (Figure 4E) that VIM-HA-full length protein was distributed in both cytoplasm and nucleus of HeLa cells, and there was obvious co-localization with PGRMC1 protein, in which the protein N-fragment had obvious intranuclear co-localization with PGRMC1 protein, while F-fragment protein has cytoplasmic co-localization with PGRMC1 protein.

The above experimental results indicate that PGRMC1 can bind to intracellular VIM and may further affects the function of VIM.

### 3.4. PGRMC1 Regulates the Phosphorylation Level at the Ser-39 Site of VIM

**Figure 5:**
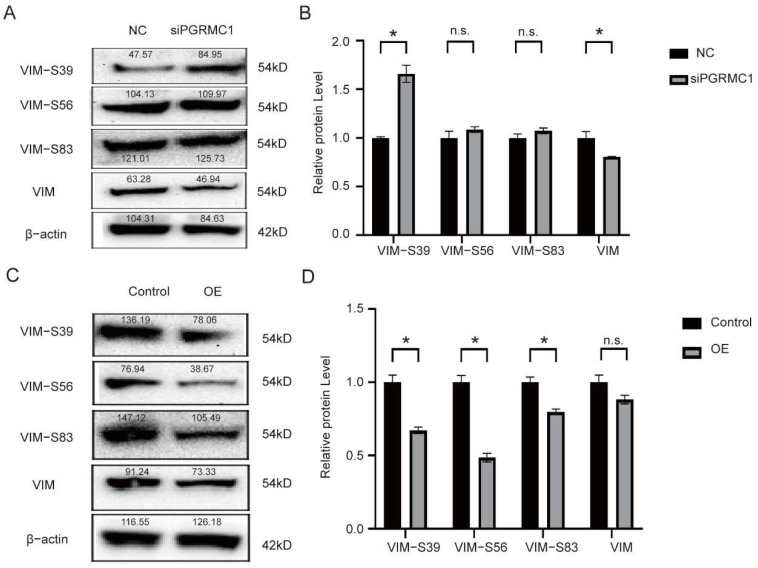
The effect of inhibiting and overexpressing PGRMC1 on the phosphorylation of VIM. (**A**) Western blot was used to analyze protein levels of VIM, VIM-Ser39, VIM-Ser56, VIM-Ser83 and β-actin in HeLa cells transfected with PGRMC1 siRNA or negative control siRNA, the data are normalized to the β-actin control; (**B**) The gray-scale processing result of (**A**); (**C**) Western blot was used to analyze protein levels of VIM, VIM-Ser39, VIM-Ser56, VIM-Ser83 and β-actin in HeLa cells transfected with PGRMC1 plasmid or vector, the data are normalized to the β-actin control, OE: overexpression; All data are from three independent experiments. (**D**) The gray-scale processing result of (**C**). * = *p* < 0.05, n.s. = not significant. Data are mean ± SD.

Among the multiple VIM phosphorylation sites, we focused on 3 major sites (Ser-39, Ser-56, Ser-83). Western blot experiments were performed in HeLa cells, and the cells were divided into experiments into the interference with PGRMC1 group (siRNA group) and the control group (control) for the protein expression validation of the three phosphorylation sites, the results of protein expression were analyzed using gray-scale analysis, and the results showed: the expression level of VIM-S39 was significantly up-regulated, while the expression level of VIM was significantly down-regulated (Figure 5A,B):

To further verify the impact of siPGRMC1 on VIM phosphorylation, we overexpressed PGRMC1 in HeLa cells to determine whether high levels of PGRMC1 affect VIM phosphorylation. And the results showed: after transfecting with PGRMC1 plasmid, the expression levels of VIM-39, VIM-S56 and VIM-S83 were all significantly downregulated (Figure 5C,D). From the above experiments, we concluded that PGRMC1 could bind to the VIM Ser-39 site and inhibit its phosphorylation, making VIM unable to depolymerize, thus promoting invasive cell migration.

### 3.5. The Protein Level of VIM Is Increased During the Process of CIN-CSCC Transition

**Figure 6:**
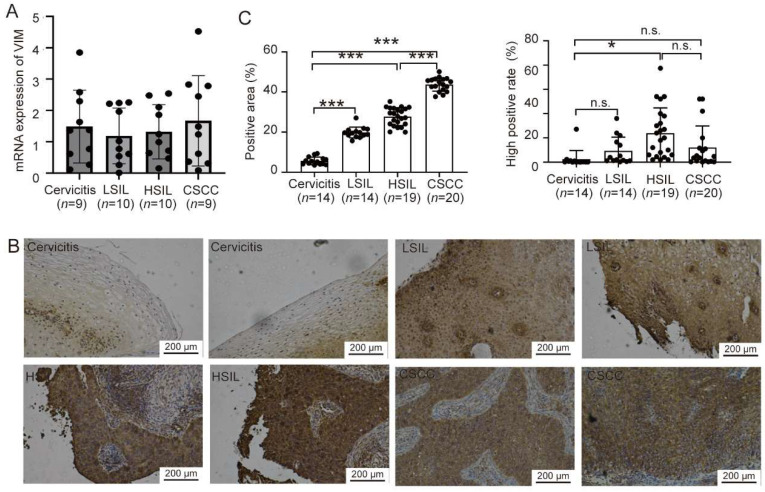
The expression changes in VIM during the process of cervical intraepithelial neoplasia. (**A**) qPCR was used to analyze mRNA expression of VIM in cervical tissues; (**B**) VIM immunohistochemical staining in cervical tissues (n = 5). (**C**) The positive area and high positive rate of VIM expression in cervical tissues related to (**B**). * = *p* < 0.05, *** = *p* < 0.001, n.s. = not significant. Data are mean ± SD.

To ascertain whether the expression pattern of VIM mirrors that of PGRMC1 in cervical tissue samples, we gathered cervical tissues at various lesion stages. These samples were categorized into four groups: Cervicitis, LSIL, HSIL, and CSCC, each containing 10 cases. We then measured the VIM expression levels in these tissue samples using qPCR. The VIM analysis results revealed that there were no significant differences in VIM mRNA expression levels among the Cervicitis, LSIL, HSIL, and CSCC groups, which simplified the comparison between these groups (Figure 6A). To further investigate, we selected samples for immunohistochemistry to assess VIM protein expression during the progression of cervical cancer lesions. The analysis of positivity rates indicated that the area showing positive VIM staining progressively increased from the Cervicitis group to the CSCC group. This trend suggested that PGRMC1-positive cells gradually dominated the cervical tissue during the transition from CIN to CSCC, paralleling the behavior observed with PGRMC1. Furthermore, when compared to the Cervicitis group, the HSIL tissues exhibited a significantly higher rate of high VIM positivity. Although the CSCC group appeared to have a lower high positivity rate than the HSIL group, the difference was not statistically significant. There was also no significant difference in the high positivity rate between the Cervicitis and CSCC groups (Figure 6B,C). Thus, our results suggested that the VIM protein expression not its RNA expression is increased during the process of CIN-CSCC transition, which was similar to PGRMC1.

## 4. Discussion

In this experiment, employing a series of bioinformatics analyses and molecular-protein level investigations, we discovered that PGRMC1 inhibits the depolymerization of VIM by binding to its Ser-39 phosphorylation site, thereby preventing VIM phosphorylation. This mechanism subsequently enhances the migratory and invasive capabilities of HeLa cells. Consequently, our findings indicate that PGRMC1 exerts a promotional effect on cervical intraepithelial neoplasia, establishing it as a significant poor prognostic factor for cervical cancer due to its role in facilitating tumor migration and invasion.

Vimentin (VIM), a critical member of the type III intermediate filament protein family, is a highly conserved and multifunctional cytoskeletal protein essential for maintaining cellular morphology, transmitting mechanical stress, and facilitating signal transduction. Encoded by one of the largest gene families of intermediate filament proteins, VIM is a key structural component of the cytoskeleton. It plays a pivotal role in tissue fibrosis, wound healing, and is extensively involved in physiological processes such as inflammatory responses and lipid metabolism [12]. Recent studies have indicated that VIM plays a pivotal role in tumorigenesis, development, and metastasis, emerging as a novel therapeutic target for various tumors [13]. Within the realm of gynecologic oncology research, investigations into VIM expression and its function are relatively nascent. Existing research has demonstrated that VIM expression levels are notably higher in cervical cancer tissues compared to those in chronic cervicitis, low-grade cervical intraepithelial neoplasia (CIN), and high-grade CIN. Additionally, the methylation degree of its promoter region increases, suggesting that VIM could serve as a significant molecular marker for the progression and infiltration of cervical cancer [14]. In our experiment, through immunohistochemical analysis of collected typical clinical samples, we observed that VIM expression levels gradually increased and exhibited a significant trend during the process of cervical intraepithelial neoplasia. This finding also suggests that the elevation of VIM may serve as a potential indicator for cervical cancer.

At the molecular level, the biological function of Vimentin (VIM) is predominantly regulated through phosphorylation modifications. Key phosphorylation sites identified to date include Ser39, Ser56, and Ser83, among others. VIM features multiple phosphorylation sites, each modulated by distinct kinases, primarily protein kinase C (PKC), cell-cycle-dependent kinase 1 (CDK1), mitogen-activated protein kinase (MAPK), casein kinase 2 (CK2), and others. Phosphorylation at Ser39 is intricately linked to the in vivo assembly of VIM intermediate filaments. This modification induces the depolymerization of VIM fibers, thereby remodeling the cytoskeletal structure and altering the cell’s morphology and mechanical properties. VIM-Ser56 undergoes phosphorylation by PKC and PAK, leading to the rearrangement of the intermediate filament network. This rearrangement provides a structural foundation for cell deformation and migration [15]. The VIM-Ser83 site is phosphorylated by CDK1, which triggers the rearrangement and depolymerization of the VIM fiber network. This process is crucial for the cell’s entry into mitosis, chromosome segregation, and cell division [16]. Conversely, the VIM-S39 site is phosphorylated through AKT-mediated pathways and is recognized as a phosphorylation site specific for certain kinases. This site can bind to various phosphorylated kinases, activating protein phosphorylation and thereby enhancing the migratory and invasive capabilities of cancer cells [17]. In an organoid co-culture model of colorectal cancer, an upregulation in the phosphorylation level of VIM is considered a marker of epithelial–mesenchymal transition (EMT) and is associated with the invasive phenotype induced by stromal cells. This model simulates signal transduction within the tumor microenvironment, promoting the migration and invasion of tumor cells [18]. Phosphorylation proteomics data reveal that in FAT1-mutated small cell lung cancer, 24 phosphorylation sites of VIM exhibit significant increases. These sites are primarily involved in cytoskeletal recombination and cell motility processes, indicating a close relationship between VIM phosphorylation and the metastatic potential of small cell lung cancer [19]. The VIM plays a pivotal role in promoting the directional migration of cells in organoids by regulating cytoskeletal stability. In breast cancer cells, overexpression of Vimentin enhances cell hardness, motility, and directional migration ability, reorients microtubule polarity, and increases the EMT phenotype. These findings suggest that Vimentin maintains the mechanical integrity of EMT cancer cells by regulating cytoskeletal structure, thereby facilitating tumor metastasis. As an intermediate filament protein within the cytoskeleton, Vimentin interacts with stress fibers, influencing their dynamic flow and facilitating the transport of endocytic vesicles. This interaction plays a crucial role in the process of macrophages degrading the extracellular matrix (ECM), a process closely associated with tumor invasion [20].

PGRMC1 exhibits high expression levels in breast and ovarian cancer tissues, where it is closely associated with malignant biological behaviors, including tumor cell proliferation, migration, and invasion. In breast cancer cells, PGRMC1 interacts with estrogen receptor α (ERα), activating the ERα signaling pathway and thereby promoting cancer cell proliferation. Similarly, in ovarian cancer, PGRMC1 enhances cell proliferation and migration through the activation of the ERK1/2 signaling pathway. Given its pivotal role, PGRMC1 emerges as a promising drug target. As a targetable protein and a component of a multi-protein complex, it binds progesterone, other steroids, and various drug compounds. Its ability to bind heme further underscores its potential in drug development. Designing drugs that specifically target PGRMC1 could inhibit tumor cell survival and proliferation [21]. However, the development of targeted drugs against PGRMC1 presents significant challenges. Despite its attractiveness as a therapeutic target, PGRMC1’s complex interactions with other proteins and diverse functions complicate the design and development process. To date, no mature PGRMC1-targeted therapeutic drugs have received approval for clinical use. Additionally, research on the upstream and downstream regulatory factors of PGRMC1 in tumor cells remains limited, and its specific signal transduction network in hormone replacement therapy has not been fully elucidated. In our Western blot experiments, we successfully demonstrated that PGRMC1 influences the phosphorylation level at the S39 site by binding to the VIM-39 domain, thereby mediating protein–protein interactions. We hypothesize that the binding of PGRMC1 to the VIM-S39 domain may exert an antagonistic effect, promoting tumor cell migration and invasion, as well as the development of cervical intraepithelial neoplasia, by occupying the phosphorylation activation site on VIM and inhibiting phosphorylation at the VIM-S39 site. Nevertheless, the precise mechanisms underlying these processes remain to be thoroughly investigated.

There are some limitations of this study to point. In the bioinformatics analysis section, although we performed prognostic survival analysis basing on public databases (the TCGA database) and pointed that PGRMC1 is a poor prognostic factor; however, we do not have independent cohorts to verify this result. Furthermore, the clinical sample size remains relatively small. In the Co-IP experiments, although we demonstrated binding between PGRMC1 and VIM, but whether PGRMC1 directly binds to VIM is unclear. Future studies will be made up for these limitations and to clarify the underlying function of PGRMC1 in CIN-CSCC transition.

To sum up, our study finds that PGRMC1 expression is elevated in the process of CIN-CSCC transformation, and the specific mechanism is that PGRMC1 binds to VIM protein, inhibits the phosphorylation level of VIM (Ser-39) site to maintain VIM assembly, and interferes with PGRMC1 expression to inhibit cervical cancer cell migration and invasion. This study provides a new target for the intervention and treatment of cervical squamous carcinoma, which has important clinical significance.

## 5. Conclusions

Taken together, we found that the expression level of PGRMC1 protein gradually increased during the process of cervical intraepithelial squamous change. PGRMC1 binds to VIM and inhibits the phosphorylation of VIM protein through its Ser-39 site. The lack of PGRMC1 reduces the migration and invasion of CSCC cells.

## Figures and Tables

**Table 1:** Patient characteristics.

Stage		Cervicitis (n = 10)	LISL (n = 10)	HSIL (n = 10)	CSCC (n = 10)
Age		36.8	39.5	40	58.4
HPV infection	single	5	7	6	8
	multiple	5	3	4	2
Risk *	High	10	10	10	10
	Middle	1	2	3	1
	Low	0	1	0	1

* High-risk HPV subtype: HPV16, 18, 31, 33, 35, 39, 45, 51, 52, 56, 58, 59, 68; Middle-risk HPV subtype: HPV26, 53, 66, 73, 82; Low-risk HPV subtype: HPV6, 11, 40, 42, 43, 44, 54, 61, 70, 72, 81, 89.

**Table 2:** siRNA sequence.

Gene	Primer
siPGRMC1-1	Forward 5′-GGCAAGGUGUUCGAUGUGA-3′
	Reverse 5′-UCACAUCGAACACCUUGCC-3′
siPGRMC1-2	Forward 5′-GCAUCUUCCUGCUCUACAA-3′
	Reverse 5′-UUGUAGAGCAGGAAGAUGC-3′
siPGRMC1-3	Forward 5′-GUACUCAGAUGAGGAAGAA-3′
	Reverse 5′-UUCUUCCUCAUCUGAGUAC-3′

**Table 3:** Primers sequence for qPCR.

Gene		Primer
ACTB (human)	Forward	5′-GCCGAGGACTTTGATTGC-3′
	Reverse	5′-CCTGTGTGGACTTGGGAGA-3′
PGRMC1 (human)	Forward	5′-GUACUCAGAUGAGGAAGAA-3′
	Reverse	5′-UUCUUCCUCAUCUGAGUAC-3′
VIM (human)	Forward	5′-CGTGAATACCAAGACCTGCTC-3′
	Reverse	5′-GGAAAAGTTTGGAAGAGGCAG-3′
MMP2 (human)	Forward	5′-ACCCATTTACACCTACACCAAG-3′
	Reverse	5′-TGTTTGCAGATCTCAGGAGTG-3′
MMP9 (human)	Forward	5′-CGAACTTTGACAGCGACAAG-3′
	Reverse	5′-CACTGAGGAATGATCTAAGCCC-3′

## Data Availability

The raw data supporting the conclusions of this article will be made available by the authors on request.

## Abbreviations

The following abbreviations are used in this manuscript:

CIN	cervical intraepithelial neoplasia
CSCC	cervical squamous carcinoma
HPV	human papillomavirus
LSIL	low-grade squamous intraepithelial lesions
HSIL	high-grade squamous intraepithelial lesions
PGRMC1	progesterone receptor membrane component 1
VIM	Vimentin
RFS	recurrence-free survival
OS	overall survival
DMFS	distant metastasis-free survival
